# The de winter pattern: time is myocardium

**DOI:** 10.1097/j.pbj.0000000000000311

**Published:** 2025-12-04

**Authors:** Miguel B. A. Vicente, Eric Monteiro, Domingos Ramos, Lino Gonçalves

**Affiliations:** a Cardiology Department, Coimbra Hospital and University Centre, Coimbra, Portugal; b Cardiovascular and Thoracic Centre—Clínica Girassol, Luanda, Angola; c Faculty of Medicine, University of Coimbra, Coimbra, Portugal; d Coimbra Institute for Clinical and Biomedical Research (iCBR), Coimbra, Portugal.

## Case

A 45-year-old man, a bank clerk, with no known relevant personal history, with a family history of sudden unexplained death of his father at the age of 51 and of a brother who underwent percutaneous coronary intervention at the age of 38 and coronary artery bypass surgery at the age of 48, presented to the emergency department of a district hospital with oppressive, severe chest pain, without irradiation and worsening with exertion. On objective examination, he was conscious, orientated and cooperative, and sweating. Blood pressure was 117/70 mmHg, heart rate 75 beats per minute, and peripheral oxygen saturation 97%.

**Figure 1.**
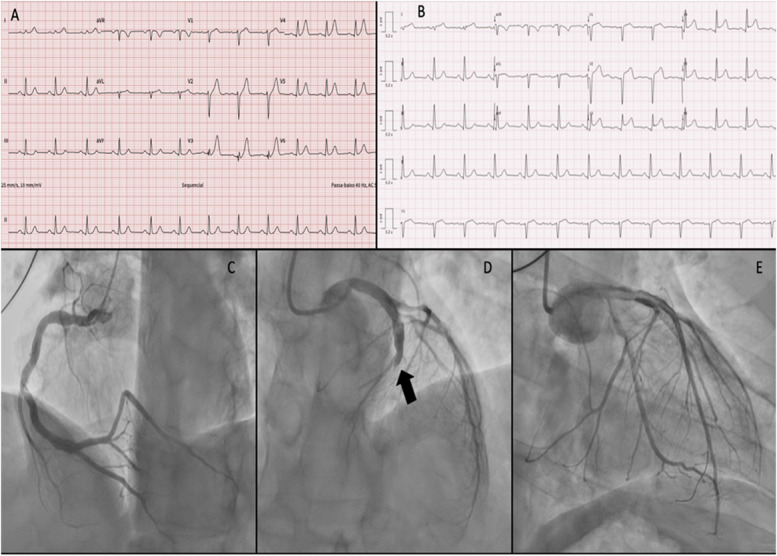
A, Electrocardiogram: de Winter's pattern: upsloping ST-segment depression and apiculate and symmetrical T wave in precordial leads (namely V2‒V6); (B) electrocardiogram on arrival, 1 hour later: acute myocardial infarction with ST-segment elevation DI-aVL, V2‒V6. Coronary angiography: (C) dominant right coronary artery, with irregularities, without angiographically significant lesions. D, left main trunk without lesions, anterior descending occluded in the middle segment (arrow). First diagonal with 75% ostial lesion. E, Angioplasty of the occlusion of the anterior descending artery with a final TIMI 3 result.

The initial electrocardiogram showed sinus rhythm, 75 beats per minute, upsloping ST-segment depression and peaked, symmetrical T waves in the precordial leads (namely V3‒V6), compatible with the de Winter pattern (Fig. 1A). Analytically, hemoglobin 14.5 mg/dL, ultrasensitive troponin 36.783,8 ng/L (reference value <34 ng/L), NT-proBNP 70 pg/mL. He was transferred to a nearby hospital with primary percutaneous coronary intervention capacity within an hour. Upon arrival, there was a change in the previously identified electrocardiographic pattern with ST segment elevation in the anterior leads (Fig. 1B). The patient was in Killip Kimball class I. Dual antiplatelet therapy was initiated with a loading dose of acetylsalicylic acid, ticagrelor, and unfractionated heparin.

The patient underwent coronary angiography, which revealed a dominant right coronary artery (Fig. 1C). An occlusion of the left anterior descending artery in the middle segment was present (Fig. 1D). Angioplasty of the culprit lesion was performed with predilation using a 2.5 × 15 mm balloon, followed by implantation of a 3.0 × 21 mm Ultimaster Nagomi drug-eluting stent (Terumo International Systems, Tokyo, Japan), with a TIMI 3 result (Fig. 1E). During hospitalization, the metabolic profile was as follows: lipoprotein (a): 118.10 mg/dL and apolipoprotein B: 133 mg/dL, total cholesterol: 253 mg/dL, high-density lipoprotein cholesterol: 44 mg/dL, low-density lipoprotein cholesterol: 188.4 mg/dL, triglycerides: 103 mg/dL, and hemoglobin: A1C 5.3%. The transthoracic echocardiogram revealed an ejection fraction of 57%, with no valvular heart disease. The patient progressed well and was discharged from hospital on the fifth day of treatment, with dual antiplatelet therapy, high-intensity statin associated with ezetimibe, and recommendations for lifestyle changes.

de Winter's pattern is an electrocardiographic alteration associated with the hyperacute phase of acute myocardial infarction,^1,2^ present in around 2–3.4% of cases of acute coronary artery occlusion, predominantly of the anterior descending artery.^3,4^ It is characterized by upsloping ST-segment depression, an apiculate and symmetrical T wave in the anterior leads.^1-4^

It is worth noting that the de Winter's pattern can be confused with other medical conditions that manifest with apiculate T waves, for example, those that occur in the context of hyperkalemia, but also as a manifestation of early ischemia, exhibiting very high T waves, with a broader base and preceded by ST-segment elevation.^3^ Unlike de Winter's pattern, which usually lasts from the first medical contact until primary percutaneous coronary intervention, hyperacute T waves appear seconds after total occlusion of a coronary artery and usually disappear within minutes.^5^

Although the exact pathophysiology has yet to be fully clarified, several hypotheses have been proposed to explain this nosological entity, the most widely accepted of which is the attribution of this phenomenon to subendocardial ischemia, which alters myocardial action potentials.^2,3^ In the hyperacute phase of the ischemic event, subendocardial potentials are more affected than subepicardial potentials, before transmural ischemia sets in. This difference in vulnerability and electrical response between the layers explains the characteristic clinical and electrocardiographic manifestations of this condition.^2^

It is essential that cardiologists and emergency physicians quickly recognize this electrocardiographic pattern so that patients can be referred immediately for coronary reperfusion. Failure to recognize this pattern can delay early intervention, resulting in negative prognostic effects associated with myocardial infarction.

We conclude that

(1) de Winter pattern is an atypical electrocardiographic pattern equivalent to acute coronary syndrome with ST-T segment elevation, usually associated with anterior descending occlusion.
(2) Its early recognition makes it possible to carry out reperfusion therapy, such as primary angioplasty or thrombolysis, in good time to reduce mortality and preserve myocardial function.
